# Protective Effect of the MCP-1 Gene Haplotype against Schizophrenia

**DOI:** 10.1155/2019/4042615

**Published:** 2019-12-02

**Authors:** Hana Saoud, Oumaima Inoubli, Sihem Ben Fredj, Mohsen Hassine, Bochra Ben Mohamed, Lotfi Gaha, Besma Bel Hadj Jrad

**Affiliations:** ^1^Laboratory of Genetics, Biodiversity and Bioresource Valorization, Higher Institute of Biotechnology of Monastir, University of Monastir, Monastir, Tunisia; ^2^Department of Epidemiology, Farhat Hached University Hospital, Sousse, Tunisia; ^3^Hematology Department, Fattouma Bourguiba University Hospital, TN 5000, Monastir, Tunisia; ^4^Department of Psychiatry and Vulnerability to Psychoses Laboratory-CHU Monastir, University of Monastir, Monastir, Tunisia

## Abstract

While cytokines and their genetic variants have been intensively studied in schizophrenia, little attention has been focused on chemokines in the last years. The monocyte chemoattractant protein 1 (MCP-1) is known to attract peripheral monocytes to the brain during an inflammatory reaction and to affect the T helper (Th) cell development by stimulating Th2 polarization. Owing to the neuroinflammation in schizophrenia and the variable level of MCP-1 in these patients' sera, we proposed to analyze the impact of functional genetic variants of the MCP-1 gene (MCP-1-2518A/G (rs1024611), MCP-1-362G/C (rs2857656), and MCP-1 int1del554-567 (rs3917887)) in schizophrenic patients. We conducted a case-control study on a Tunisian population composed of 200 patients and 200 controls using RFLP-PCR. Our results indicated that the minor alleles (-2518G and Del554-567) were significantly more prevalent in controls than in patients (*P*=0.001/adjusted OR = 0.42, *P*=0.04/adjusted OR = 0.64), whereas, for -362C minor allele, increased risk of schizophrenia was revealed (*P*=0.001, adjusted OR = 2.38). In conclusion, we have identified the haplotype combination -2581G/-362G/int1del554-567 that could mediate protection against schizophrenia (*P*=0.0038, OR = 0.19) and the effect could result more strongly from the MCP-1 -2582G with -362G variants, whereas the effect of int1del554-567 may in part be explained by its LD with -362.

## 1. Introduction

Schizophrenia (SCZ) is a chronic and disabling neuropsychiatric disease. It usually appears in the late second and third decades of life with a varied symptomatology, generally belonging to three classes, namely, positive symptoms, which include hallucinations, delusions, and thought disorganization; negative symptoms, which refer to reduced motivation and emotional vibrancy; and cognitive deficits that reflect in particular an alteration in working memory, executive functions, and attention processes [1]. The causes remain unclear, and given the heterogeneous nature of the disorder, it is likely that there are multiple contributing etiological agents. Several studies have supported the role of an inflammatory component. In line with it, increased levels of cytokines such as TNF-*α*, IL-6, IFN-*γ*, and IL-1*β* were detected in the blood and cerebrospinal fluid in the first onset and acute relapse patients with schizophrenia [2]. A postmortem study reported that the expression of a microglial gene is dysregulated [3] and aberrant functions of microglia have potential implications in this disorder [4, 5]. However, recent knowledge of the high degree of macrophage plasticity and microglia has brought to light that, in inflammatory conditions, microglial cells polarize to M1 phenotype and produce proinflammatory cytokines/mediators including IL-1*β*, IL-6, TNF-*α*, CCL2, ROS, and NO [6, 7]. This prompted a renewed interest in the participation of chemokines which are a family of small cytokines whose name is derived from their ability to induce directed chemotaxis in nearby responsive cells. In particular, analyses revealed an increased number of monocytes and elevated levels of CCL2 in the first-episode psychosis (FEP) patients and multiple-episode schizophrenia (MES) individuals [8, 9], suggesting that this chemokine may contribute to the development of this disorder.

Monocyte chemoattractant protein-1 (MCP-1), known as CCL2 (chemokine (C-C motif) ligand 2), is a small cytokine that belongs to the CC chemokine family. It is not only expressed in neuroinflammatory conditions but is also constitutively present in the brain in both glial cells [10–13] and neuron [14]. In addition to being considered as a potent mediator for the attraction of monocytes and macrophages to the sites of inflammation, MCP-1 mediates the transendothelial migration of inflammatory cells across the blood-brain barrier and modulates the local inflammatory response by forming chemotactic gradients within the CNS [15]. Variation in MCP-1 expression can be influenced by polymorphic variants of the gene, which is mapped to chromosome 17 (17q11.2-q12) [16]. Among various polymorphisms, two functional genetic variations within the MCP-1 gene promoter, rs1024611 A/G and rs2857656 G/C, were linked to variable levels of CCL2 [17, 18].

The -2518A/G (rs1024611) polymorphism is located in the distal regulatory region of the MCP-1 gene relative to the major transcriptional start site of the gene that can influence the transcriptional activity of MCP-1 and contributes to susceptibility to multiple sclerosis, Alzheimer's disease, and major depressive disorder [18–21]. The second polymorphism, MCP-1 rs2857656 (-362 G/C), has been identified in the proximal promoter region of the gene and was reported to increase the risk of spinal tuberculosis and carotid atherosclerosis by enhancing MCP-1 expression levels [17, 22]. Also, a 14 base-pair deletion in the first MCP-1 intron, int1del554-567 (rs3917887), which could affect the transcriptional activity of the gene has been analyzed [23]. Considering the role of MCP-1 in modulating immune system response together with the relationship between immune abnormalities and schizophrenia, the primary aim of this study was to investigate the potential role of these three single-nucleotide polymorphisms in conferring susceptibility to or protection against schizophrenia.

## 2. Material and Methods

### 2.1. Study Participants

The study included 200 patients hospitalized or received in out-patient services at the Psychiatry Department of Monastir Hospital, Tunisia. All of them were diagnosed according to DSM-IV (Diagnostic and Statistical Manual of Mental Disorders, the fourth version) criteria for schizophrenia [24]. The selection was based on the exclusion of schizoaffective disorder and other mental pathologies and severe physical diseases such as cancer. For the patient to be treated and to be considered as true schizophrenic, continuous signs of the disturbance must last longer than 6 months. This 6-month period must comprise at the minimum one month of active phase symptoms. Total scores and subscores for positive, negative, and general symptoms were collected. In the current work, 45.9% of patients met the diagnostic criteria for undifferentiated, 32% for paranoid, and 19.8% for disorganized subtypes of schizophrenia. The average age of the cohort was 38.71 ± 10.69 years (range 18–65 years) with 19.5% females and 80.5% males (sex ratio = 4.12) and the onset age varied from 10 to 47 years. We used the definition of Meltzer et al. [25], which equates the age at onset of schizophrenia with the first occurrence of positive psychotic symptoms (mean age of onset ± SD 25.06 ± 7.59 years).

The control group consisted of 200 healthy volunteers with a mean age of 32.71 ± 10.8 years, ranging from 18 to 65 years and a sex ratio of 3.52 (19.9% females and 70.1% males). They were recruited among the blood donors of the same Monastir Hospital, Tunisia. The main inclusion criteria for controls were as follows: being free of any psychiatric illness, nonsubstance abuse, and without a family history of psychiatric disorders.

The institutional ethics committee of the Higher Institute of Biotechnology of Monastir in Tunisia approved the research protocol, and written consent was provided by each participant or a family member before blood collection.

### 2.2. DNA Extraction and Genotyping

Genomic DNA was extracted from the whole EDTA-anticoagulated blood, according to the salting-out technique [26]. Genotyping was determined by the restriction fragment length polymorphism analysis of PCR-amplified product (RFLP-PCR). All primers were designed using genomic sequences obtained from the National Center for Biotechnology Information (http://www.ncbi.nlm.nih.gov/) and Primer-BLAST.

Details including primers and restriction enzyme with product sizes are listed in Table 1.

### 2.3. Statistical Analysis

For genetic association analysis, each polymorphism was tested for deviations from Hardy–Weinberg equilibrium in patient and healthy groups. Statistical analysis was performed using the SPSS software (version 23, Armonk, NY, USA). The distribution of genotypes or alleles of chemokine genes between cases and controls was compared by the Chi-square test (or Fisher's exact test when *n* < 5). Odds ratios (OR) and 95% confidence intervals (CI) were also calculated whenever *χ*^2^ test was significant. Differences between groups were deemed to be significant at *P* < 0.05 for all tests. To calculate the linkage disequilibrium (LD) expressed as *D*′, the Haploview version 4.2 was used and the graphical view was generated [27]. For haplotype reconstruction, the SNPStats online software (https://www.snpstats.net/start.htm) was performed.

A binary logistic regression model with categorical and quantitative independent variables was done, adjusting for the effects of age and gender through the analysis of deviance from a sequential addition of each variable.

## 3. Results

The determination of genotypic and allelic frequencies of investigating SNPs is given in Table 2. Genotype distribution was in accordance with HWE in both controls and cases for all markers.

The promoter allele -2581G was significantly associated with resistance to schizophrenia (*P*=0.000001; OR = 0.36), whereas, for -362C allele, an increased risk of schizophrenia was revealed (*P*=0.001; OR = 1.58). There was no statistically significant difference between the patient and the control groups in terms of allele frequency of the MCP-1 int1del554-567 polymorphism (*P*=0.075; OR = 0.7). A strong association was seen for both heterozygous and homozygous carriers of MCP-1 -2518G (OR = 0.35, *P*=0.000015; OR = 0.1, *P*=0.015, respectively). Differently, only the frequency of MCP-1 -362 (CC) minor homozygote genotype was shown to be significantly more prevalent among healthy controls (OR = 2.33, *P*=0.001). Yet, the CCL2I/D heterozygous genotype frequency was found to be higher in patients (OR = 0.61, *P*=0.03).

These associations remain the most highly significant with the lowest Akaike information criterion (AIC) according to the dominant model of inheritance for -2518A/G and int1del554-567 polymorphisms and to the recessive model of inheritance for the -362G/C polymorphism (Table 2).

As a way to boost statistical power, phenotypic parameters were correlated with the genetic data. Thus, we classified schizophrenic patients into cases with and without specific characteristics to give a robust reduction in the heterogeneity of this pathology. When we stratified the studied populations, according to their gender, our results showed that the -2518G allele frequency was higher in male controls (OR = 0.43, *P*=0.001) but the -362C allele frequency was increased among male patients (OR = 1.79, *P*=0.0004). Similar results have been found for females without reaching the statistical significance, potentially related to the small size of the female population analyzed (OR = 0.7, *P*=0.13; OR = 1.13, *P*=0.7, respectively). However, for the int1del554-567 Del, allele differences were not significant for both sexes (Males: OR=0.7, *P*=0.13, Females: OR=0.44, *P*=0.06).

Furthermore, when patients were stratified on the basis of clinical subtypes, genotype and allele frequencies of the SNP at position-2518 were higher in healthy individuals regardless of the three schizophrenia forms. Concerning the -362G/C polymorphism, the association remained significant with paranoid and undifferentiated schizophrenia but not with the disorganized type of the disease. The genetic distribution of the MCP-1 int1del554-567 polymorphism between the studied groups did not reveal any association with the different clinical forms. To control the possible bias of potentially confusing confounders, a logistic regression model including sex, age, and genotype variables, conforming to the appropriate genetic model of inheritance, showed that the mutated genotypes MCP-1 -2518 (AG + GG), MCP-1 -362 (CC), and MCP-1 int1del554-567 (Ins/Del + Del/Del) were correlated with schizophrenia (*P*=0.001/adjusted OR = 0.42, *P*=0.001/adjusted OR = 2.38, and *P*=0.04/adjusted OR = 0.6, respectively) and the same result was confirmed for the association with the different clinical forms.

**Figure 1:**
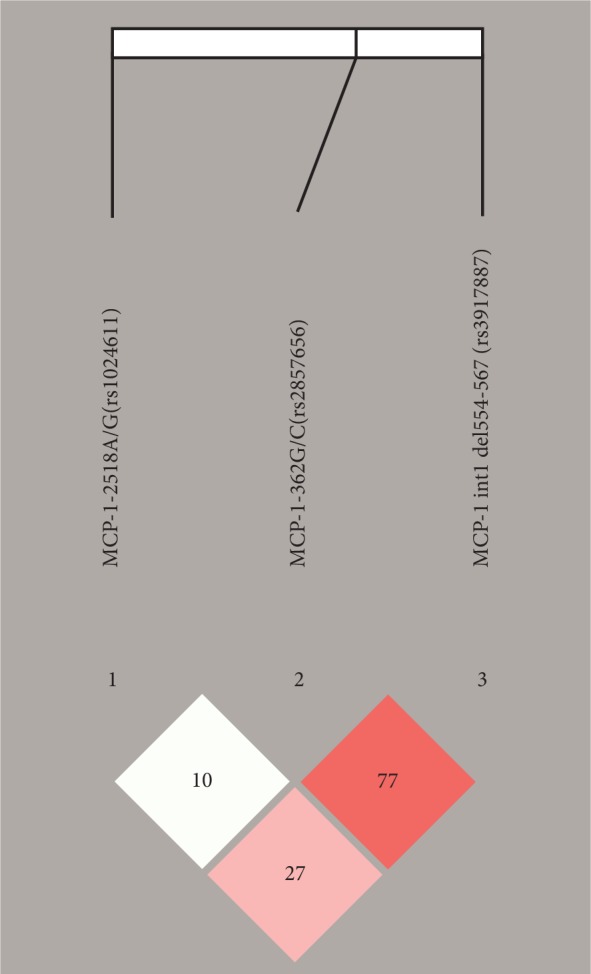
Linkage disequilibrium (LD) map of the three MCP-1 SNPs genotyped using Haploview. The positions of the tested variants are indicated above the Haploview output. The LD between specific pairs of MCP-1 SNPs is displayed by the color scheme, which represents LD relationships, based on *D*′ values (linkage coefficient according to Lewontin) multiplied by 100. Bright red squares illustrate high LD, squares in shades of pink/red reveal moderate LD, and white squares indicate no statistically significant difference of LD.

Haploview analysis revealed strong LD between rs2857656 and rs3917887 (*D*′ = 77) as compared to that conferred by rs1024611 with rs3917887 (*D*′ = 27) and rs2857656 (*D*′ = 10) (Figure 1).

Only haplotypes having a frequency of more than 5% were considered. Statistically significant differences in frequencies between cases and controls were observed in one out of the four combinations present (G-G-Ins) containing the mutated G allele of the rs102411. The G-G-Ins haplotype was associated with protection against schizophrenia as it was higher among healthy subjects compared to patients (12.96% vs 2.48%). Estimated haplotypes constructed for the three markers in both patients and controls are presented in Table 3.

## 4. Discussion

Dysregulations of the immune system are being increasingly studied and seem to play a major role in the pathophysiology of schizophrenia (SCZ). In line with it, chemokine genes have been candidates for the etiology of this disorder based on their role in driving inflammation and immune responses and regulating many neuronal functions [28].

Our results showed that three polymorphisms, regulating positively the expression of the CCL2 chemokine, could have a protective effect on the schizophrenia susceptibility. Indeed, the higher prevalence of the MCP-1 -2518G allele in controls as compared to SCZ patients in the current study suggests that the minor G allele acts as a protective factor against SCZ. Our findings go in the same direction as those reported in the Korean population showing that the G allele frequency is significantly lower in major depressive disorder [19] and tends toward significance in schizophrenia with positive symptomatology [29]. But the contradiction in the previous reports indicated no significant association between any allele and SCZ in Italian [30] and Turkish [31] populations nor the recent study in an Armenian sample [32] which found that the G allele dominantly conferred disease susceptibility.

First, we verified that the genetic frequencies identified in the controls of our study were similar to those reported in other Tunisian studies analyzing different pathologies, which eliminates the possibility of handling errors [33–35]. Also, the discrepancies cited above cannot be only explained by diverse racial and ethnic origins. We noted that the genetic frequencies of controls that were found in our study are similar to those shown in Italian [30], Turkish [31], and Armenian [32] population but differed a lot from those described in the Korean population [19, 29]. The disparity in the results might rather be explained by the inclusion criteria, that is, small sample size and the individual-level factors including lifestyle, dietary variations, and environmental variables. For instance, cases consisted of one hundred and three and they were all with paranoid schizophrenia in the Armenian study [32]. This clinical former represents only 32% of patients in our work. Concerning the Italian study [30], 35 out of 191 subjects met the criteria for schizoaffective and catatonic disorders, which were excluded in our selection.

For MCP1 -2581G, several studies have demonstrated increased gene expression in vitro and elevated MCP-1 plasma levels in vivo [18, 36–38]. Moreover, a Tunisian study reported that CCL2 plasma concentrations were higher in both asthmatic patients and controls carrying the G allele than in subjects with A polymorphism [39], and these suggest that the G allele could correlate with a higher level of MCP-1 in our Tunisian general population.

We also report, for the first time, a similar genetic correlation between the proximal -362 G/C SNP and schizophrenia where the frequency of the wild G allele is higher in the controls compared to the patients suggesting its protecting effect. Results of the reporter gene assay showed that both variants, MCP1 -362G and Ins554-567, exert a higher transcriptional activity, eventually resulting in higher production of MCP-1 [40]. It can be suggested that the -362G allele could induce a protective effect against schizophrenia via a production of higher level of MCP-1. In connection with these previous results, the haplotypic analysis showed that the -2581G/-362G/INS haplotype carrying the two alleles associated with higher production of MCP-1 conferred the highest significant difference (OR = 0.19, CI 0.06–0.59, *P*=0.0038) compared to the wild-type combination. Haplotype distribution profile suggests that the observed protection status may be driven more strongly by the MCP-1 -2518G allele with -362G variants, whereas the effect of int1del554-567 may in part be explained by its LD with -362.

As far as we know, this is the first report of these three variants being examined for association with SCZ. This correlation persisted after adjusting data for confounding factors using logistic regression. The pleiotropic actions of this chemokine on the CNS could explain its involvement in the pathophysiology of schizophrenia. First, the beneficial property of CCL2 in the brain could result from its known role in Th2 polarization. Indeed, higher concentrations of MCP-1 can suppress IFN-*γ* [41] and IL-12p40 production, a common component of IL12 and IL23 [36, 42], and upregulate IL4, the differentiation of Th0 cells into Th2 cells in the peripheral lymphoid organs. It results in a Th2 immunological reaction predominance and underactive the Th1 and/or Th17 immunological reaction [43]. These findings support the Th1/Th2 immune system imbalance hypothesis in schizophrenia, suggesting that Th1 response increases the schizophrenia susceptibility [2, 44].

Second, it was shown that CCL2 has the capacity to modulate the release of some brain neurotransmitters. It was revealed that CCL2 enhances AMPA and NMDA receptor currents in spinal neurons and modulates via a presynaptic effect glutamate secretion [45]. In addition, a strong decrease of the transcripts related to glutamatergic neurotransmission and dysregulation of glutamatergic pathways in schizophrenic patients were provided by using DNA microarray techniques [46, 47] and functional neuroimaging studies [48, 49].

Third, the extensive and dynamic expression of CCL2 during in utero development suggests a crucial role of this chemokine in the neurogenesis process as promoting the growth of dendrites and synapses [50]. Specifically, numerous chemokines including CCL2 have been involved in the regulation of neural stem/progenitor cell migration, proliferation, differentiation, and integration of new neurons into functional circuits [51]. Implicitly, altered expression in this chemokine network could induce a disruption of early neurodevelopment which has been implicated in many psychiatric disorders, particularly schizophrenia where a number of prenatal maternal environmental factors have been proposed as risk factors for the development of this disorder [52–54]. The levels of MCP-1 were investigated in schizophrenia. Findings are equivocal with nearly equal numbers of studies showing significantly higher [8, 55, 56] or similar [57, 58] levels between schizophrenia patients and healthy comparison subjects (HCs) probably linked to the heterogeneity of clinical features of patients. These results and the high-lighted correlation of MCP-1 genetic polymorphism with positive symptoms scale strengthen the involvement of MCP-1 in the schizophrenia development.

In conclusion, our results identified the haplotype combination -2581G/-362G/int1del554-567 that could mediate protection against schizophrenia in the Tunisian population. These findings are to be extended by additional studies, including the monitoring of the MCP-1 protein levels in longitudinal studies, which can help to assess the functional significance of the observed preventing genotypes and haplotypes to schizophrenia.

## Figures and Tables

**Table 1:** Primers, fragment patterns, and enzymes used for RFLP analysis.

Polymorphisms	Primers (5′–3′)	Amplicon sizes (bp)	Restriction enzymes
-2518A/G (rs1024611)	**F**: GCTCCGGGCCCAGTATCT **R**: ACAGGGAAGGTGAAGGGTATGA	182,54 236	*PvuII*

362C/G (rs2857656)	**F**: CTAGGCTTCTATGATGCTAC **R**: TCCATTCACTGCTGAGACCA	201,110 311	*Hpy188I*

CCL2I/D (rs3917887)	**F**: GCTGATCTTCCCTGGTGCTGAT **R**: CATTAAATCCCAGTGCTTCTGCCTA	I:202 D:188	—

F: forward primer; R: reverse primer; I: insertion; D: deletion. In order to ratify the data generated by the PCR-RFPL method, we analyzed 25% of the samples randomly.

**Table 2:** Genotype distribution and allele frequencies of MCP-1 polymorphisms in healthy controls and patients with Schizophrenia.

Genotype	Healthy controls *n* = 200 (%)	Patients *n* = 200 (%)	OR (95% CI)	*P* value	AIC
MCP-1 -2518A/G (rs1024611)					
Genotypes					
AA	124 (62%)	166 (83%)			
AG	69 (34.5%)	33 (16.5%)	0.35 (0.22–0.57)	0.000015^*∗*^	
GG	7 (3.5%)	1 (0.5%)	0.10 (0.004–0.7)	0.015^*∗*^	536.4
Dominant (AA vs AG + GG)			0.33 (0.20–0.53)	0.0000023^*∗*^	536^*∗*^
Recessive (AA + AG vs GG)			0.13 (0.006–0.9)	0.067	553.4
HWE^a^	0.48	0.22			
Alleles					
A	317 (79.25%)	365 (91.25%)			
G	83 (20.75%)	35 (8.75%)	0.36 (0.23–0.55)	0.000001^*∗*^	
MCP-1 -362 G/C (rs2857656)					
Genotypes					
GG	67 (33.5%)	46 (23%)			
GC	92 (46%)	88 (44%)	1.3 (0.86–2.24)	0.17	
CC	41 (20.5%)	66 (33%)	2.33 (1.36–4.03)	0.001^*∗*^	552.4
Dominant (GG vs GC + CC)			1.68 (1.08–2.63)	0.02^*∗*^	554.8
Recessive (GG + GC vs CC)			1.9 (1.21–3.01)	0.004^*∗*^	552^*∗*^
HWE^a^	0.82	2.46			
Alleles					
G	226 (56.5%)	180 (45%)			
C	174 (43.5%)	220 (55%)	1.58 (1.2–2.09)	0.001^*∗*^	
MCP-1 int1del554-567 (rs3917887)					
Genotypes					
Ins/Ins	136 (68%)	154 (77%)			
Ins/Del	59 (29.5%)	41 (20.5%)	0.61 (0.38–0.97)	0.03^*∗*^	
Del/Del	5 (2.5%)	5 (2.5%)	0.88 (0.23–3.3)	0.8	553.1
Dominant (Ins/Ins vs Ins/Del + Del/Del)	—	—	0.63 (0.4–0.98)	0.04^*∗*^	551.4^*∗*^
Recessive (Ins/Ins + Ins/Del vs Del/Del)	—	—	1 (0.26–3.77)	>0.99	555.7
HWE^a^	0.22	1.23			
Alleles					
Ins	331 (82.75%)	349 (87.25%)			
Del	69 (17.25%)	51 (12.75%)	0.7 (0.47–1.03)	0.075	

OR: odds ratio; CI: confidence interval; HC: healthy controls; ^*∗*^*P* < 0.05; HWE: Hardy–Weinberg equilibrium; ^a^Chi-square value AIC (Akaike information criterion) provides a means for model selection.

**Table 3:** Haplotype analysis of MCP-1 polymorphisms in patients with schizophrenia and control subjects.

CCL2 haplotypes	Percent haplotype frequencies		OR (95% CI)	*P* value
	SCZ cases	HC		
A-G-Ins	41.6	37.86	1	—
A-C-Ins	40.82	28.09	1.28 (0.86–1.91)	0.23
G-G-Ins	2.48	12.96	0.19 (0.06–0.59)	0.0038^*∗*^
A-C-Del	7.91	7.38	1.00 (0.49–2.03)	1

^*∗*^ *P* < 0.05.

## Data Availability

No dataset were used to support this study.
